# Supplementary material for: Assessing the role of peer education in improving clinical and patient-reported outcomes in adults with chronic kidney disease: A scoping review protocol
Source: PLoS One. 2026 Feb 17;21(2):e0342126. doi: 10.1371/journal.pone.0342126 (PMC12912584; doi:10.1371/journal.pone.0342126)
Supplement: S2 Appendix — Search strategy. (DOCX) [file pone.0342126.s002.docx]

## S2: Appendix 1

Search strategies vary slightly to meet requirements of each database~~,~~; a draft of the APA PsychInfo search strategy is included as an example.

1. kidney diseases/
2. organ transplantation/
3. exp dialysis/
4. exp hemodialysis/
5. ((chronic or advanced or end-stage or end stage) adj3 (kidney or renal)).ti,ab,hw.
6. ((renal or kidney) adj3 (fail*)).ti,ab,hw.
7. ((renal or kidney) adj3 (transplant* or graft*)).ti,ab,hw.
8. (dialysis or predialysis or hemodialysis or haemodialysis).ti,ab,hw.
9. 1 or 2 or 3 or 4 or 5 or 6 or 7 or 8
10. Exp peers/
11. Exp peer counselling/
12. Exp peer tutoring/
13. Exp peer support/
14. Exp peer education/
15. ((peer* or peers*) adj3 (support* or educat* or tutor* or mentor* or program* or counsel*)).ti,ab,hw.
16. "peer to peer".ti,ab,hw.
17. expert patient*.ti,ab,hw.
18. "patient to patient".ti,ab,hw.
19. "peer-led".ti,ab,hw.
20. 10 or 11 or 12 or 13 or 14 or 15 or 16or 17 or 18 or 19
21. 9 and 18
22. limit 19 to yr="2000 -Current"
