# Supplementary material for: Assessing the role of peer education in improving clinical and patient-reported outcomes in adults with chronic kidney disease: A scoping review protocol
Source: PLoS One. 2026 Feb 17;21(2):e0342126. doi: 10.1371/journal.pone.0342126 (PMC12912584; doi:10.1371/journal.pone.0342126)
Supplement: S3 Table — Draft extraction form. (DOCX) [file pone.0342126.s003.docx]

## S3 **Table** 3**: Draft data extraction form**

| **General information** |
| --- |
| Researcher performing data extraction |
| Date of data extraction |
| Identification features of the study: |
| Record number (to uniquely identify study) |
| Author |
| Article title |
| Citation |
| Type of publication (e.g. journal article, conference abstract) |
| Country of origin |
| Source of funding |
| **Study characteristics** |
| Aim/objectives of the study |
| Study design |
| Study inclusion and exclusion criteria |
| Recruitment procedures used (e.g. details of randomisation, blinding) |
| Unit of allocation (e.g. participant, GP practice, etc.) |
| **Participant characteristics** |
| Characteristics of participants at the beginning of the study e.g. |
| Age |
| Gender |
| Ethnicity |
| Socio-economic status |
| Disease characteristics |
| Co-morbidities |
| Number of participants in each characteristic category for intervention and control  group(s) or mean/median characteristic values (record whether it is the number  eligible, enrolled, or randomised that is reported in the study) |
| **Intervention and setting** |
| Setting in which the intervention is delivered |
| Description of the intervention(s) and control(s) |
| **Outcome data/results** |
| Unit of assessment/analysis |
| Statistical techniques used |
| For each pre-specified outcome: |
| Measurement tool or method used |
| Unit of measurement (if appropriate) |
| Length of follow-up, number, and/or times of follow-up measurements |
| For all intervention group(s) and control group(s): |
| Number of participants enrolled |
| Number of participants included in analysis |
| Number of withdrawals, exclusions, lost to follow-up |
| Summary outcome data e.g. |
| Dichotomous: number of events, number of participants |
| Continuous: mean and standard deviation |
| Type of analysis used in study (e.g. intention to treat, per protocol) |
| Results of study analysis e.g. |
| Dichotomous: odds ratio, risk ratio and confidence intervals, p-value |
| Continuous: mean difference, confidence intervals |
| Additional outcomes - Record details of any additional relevant outcomes reported |
| Costs |
| Resource use |
| Adverse events |
