# Supplementary material for: Assessing the role of peer education in improving clinical and patient-reported outcomes in adults with chronic kidney disease: A scoping review protocol
Source: PLoS One. 2026 Feb 17;21(2):e0342126. doi: 10.1371/journal.pone.0342126 (PMC12912584; doi:10.1371/journal.pone.0342126)
Supplement: Attachment — Submitted filename: PONE-D-25-34226_comments.docx [file pone.0342126.s004.docx]

**Assessing the role of peer education in improving clinical and patient-reported outcomes in adults with chronic kidney disease: a scoping review protocol**

This is an interesting protocol that addresses an important issue in chronic kidney disease care. The idea of using peer education to support decision-making and improve patient outcomes is timely and relevant. The manuscript is generally clear and well-structured, but there are several areas where more detail and clarification would strengthen its contribution.

The scope of the review feels somewhat narrow and UK-centric, particularly with the emphasis on the ACE program. It would be helpful to explain how the findings might be generalizable to other healthcare systems or cultural contexts. At present, the global relevance and potential for broader application are not fully clear.

While the manuscript distinguishes between peer education, peer support, and peer mentoring, the inclusion of all three in the search strategy could create conceptual ambiguity. A clearer operational definition for each term and an explanation of how overlapping interventions will be handled in synthesis would improve clarity.

The methodology is well aligned with JBI and PRISMA-P guidance, but some details need elaboration. For example, the search strategy is described in general terms, yet the rationale for including or excluding certain keywords is not fully explained. How will sensitivity and specificity be balanced to avoid retrieving large volumes of irrelevant studies?

The manuscript mentions that quality will be “critically appraised,” but does not specify which tool or criteria will be used. Providing this information would enhance transparency and rigor.

Outcome measures are another area that could be strengthened, while heterogeneity is anticipated, a preliminary framework for classifying outcomes (clinical, patient-reported, decision-making quality) would help guide synthesis.

The discussion section could do more to situate this review within the broader literature on patient engagement and shared decision-making in CKD. Linking the work to global efforts or similar reviews in other chronic conditions would highlight its relevance and originality.

The manuscript mentions variations by ethnicity and socio-economic status but does not explain how these will be analysed or reported. Even if subgroup analysis is not feasible, a plan for descriptive reporting would be valuable.

Overall, this is a promising protocol that addresses an important gap. Strengthening the conceptual framework, clarifying methodological details, and emphasizing generalizability and policy implications will significantly improve its scientific merit and practical utility.
